# Auswirkungen einer Pandemie auf Menschen mit Seltenen Erkrankungen und Empfehlungen zur Aufrechterhaltung von Versorgung und Teilhabe

**DOI:** 10.1007/s00103-023-03810-4

**Published:** 2023-11-29

**Authors:** David Zybarth, Maja Brandt, Christine Mundlos, Laura Inhestern

**Affiliations:** 1 Institut und Poliklinik für Medizinische Psychologie, Universitätsklinikum Hamburg-Eppendorf, Martinistr. 52, 20246 Hamburg, Deutschland; 2 Allianz Chronischer Seltener Erkrankungen (ACHSE) e. V., Berlin, Deutschland

**Keywords:** COVID-19, Corona, Orphan Disease, Psychosoziale Unterstützung, Versorgungsforschung, COVID-19, Corona, Orphan disease, Psychosocial support, Healthcare research

## Abstract

**Hintergrund:**

Seltene Erkrankungen sind häufig durch komplexe Beschwerdebilder charakterisiert und erfordern in der Regel im Diagnose- und Versorgungsverlauf die Koordination multiprofessioneller Behandlungsteams. Im Zuge der COVID-19-Pandemie veränderten sich die medizinische Versorgung und der Lebensalltag von Menschen mit einer Seltenen Erkrankung bzw. den Eltern bei minderjährigen Patient:innen drastisch. Das Projekt RESILIENT-SE-PAN („Retrospektive Analyse der Versorgungssituation und des Lebensalltags von Menschen mit Seltenen Erkrankungen während einer Pandemie und Ableitung von Handlungsempfehlungen“) hatte auf Grundlage verschiedener Perspektiven das Ziel, die Situation von Menschen mit einer Seltenen Erkrankung während der Pandemie zu erfassen und Empfehlungen abzuleiten.

**Methoden:**

Es wurden quantitative und qualitative Befragungen aus Perspektive der Betroffenen bzw. der Angehörigen bei minderjährigen Patient:innen (i. d. R. Elternteil) und aus Perspektive von Vertreter:innen der Patient:innenorganisationen sowie ein Workshop durchgeführt (Mixed-Methods-Studie).

**Ergebnisse:**

Es zeigten sich Auswirkungen auf die Versorgung und auf den Lebensalltag sowie psychische Belastungen der Befragten. Darüber hinaus wurden COVID-19-spezifische Aspekte, Unterstützungsbedarf und auch positive Aspekte berichtet. Auf Basis der umfassenden Ergebnisse wurden insgesamt 21 Empfehlungen in den folgenden 7 Bereichen formuliert: medizinische Diagnostik und Versorgung der Seltenen Erkrankung; ergänzende Therapien, Hilfs- und Heilmittel; Zugang zu Informationen und Impfungen bzgl. COVID-19; psychosoziale Unterstützung; Alltagshilfen/Teilhabe; Patient:innenorganisationen und Sonstiges/übergeordnete Aspekte.

**Diskussion:**

Die formulierten Empfehlungen geben für künftige Krisen oder Pandemien wichtige Impulse, um die Situation von Menschen mit einer Seltenen Erkrankung adäquat zu berücksichtigen und entsprechend den Bedarfen der Betroffenen zu handeln.

## Abstract (en)

**Background:**

Rare diseases are often characterized by complex symptoms and usually require coordination of multiprofessional treatment during the diagnostic and healthcare processes. In the wake of the COVID-19 pandemic, the healthcare situation and daily life of people with rare diseases and the caregivers of children with rare diseases changed drastically. The aim of the research project RESILIENT-SE-PAN was to assess the situation of people with rare diseases and caregivers during COVID-19 and to develop recommendations based on the findings.

**Methods:**

We conducted a mixed methods study including the perspective of people with rare diseases, caregivers and representatives from patient organizations and conducted a concluding workshop.

**Results:**

The findings indicate an impact on healthcare and daily life of participants. Moreover, mental burden, supportive needs, COVID-19-specific aspects but also positive aspects were mentioned. Based on the findings from our mixed methods study, we developed 21 recommendations referring to the following topics: medical diagnostics and healthcare of the rare diseases, additional therapies and aids, access to COVID-19 information and vaccination, psychosocial support, participation and activities, patient organisations and others.

**Discussion:**

The recommendations can provide an orientation for the organisation of healthcare in future crises or pandemics in order to adequately take the situations of people with rare diseases into account and consider the needs of this patient group.

## Einleitung

Eine Erkrankung wird als selten definiert, wenn weniger als 5 von 10.000 Menschen betroffen sind (Europäische Kommission, 2008). Aktuell sind etwa 6000–9000 Seltene Erkrankungen bekannt [1, 2], wobei jährlich etwa 150–250 weitere Erkrankungen hinzukommen [1]. Es wird angenommen, dass bei etwa 3 % bis 8 % der Bevölkerung mindestens eine Seltene Erkrankung diagnostiziert wurde. Für Deutschland kann entsprechend eine Anzahl von 2,5–5 Mio. Menschen mit einer Seltenen Erkrankung geschätzt werden [2, 3].

Seltene Erkrankungen sind häufig durch komplexe Beschwerdebilder charakterisiert, welche innerhalb der Versorgung zu besonderen Herausforderungen führen können. Neben einer komplexen Diagnosestellung können im Verlauf der Aufbau und die Koordination eines multiprofessionellen, spezialisierten Behandlungsteams erforderlich sein. Die Organisation der aufgebauten Struktur kann Betroffene bzw. Eltern minderjähriger Patient:innen in einem hohen Maß belasten und im Alltag viel Raum einnehmen, insbesondere bei einer wohnortfernen Versorgung, die lange Anfahrtswege verursacht. Neben den Herausforderungen, die durch die Versorgungssituation bedingt werden, kann es auch zu finanziellen, sozialen und psychischen Belastungen kommen. So berichten Patient:innen bzw. Eltern minderjähriger Erkrankter erhöhte Depressivitätswerte, davon bis zu 42 % im klinisch relevanten Bereich [4]. Es werden Auswirkungen auf die schulische und berufliche Lebenssituation geschildert. Betroffene fühlen sich hinsichtlich diverser Bereiche (z. B. psychologische Unterstützung, Informationen) nicht ausreichend unterstützt und berichten mehr unerfüllte Unterstützungsbedürfnisse als beispielsweise Krebserkrankte [5].

Mit Beginn der Coronapandemie 2020 erfuhr der Lebensalltag aller Bürger:innen massive Veränderungen. Da Erkenntnisse zu den Folgen einer Infektion genauso wie eine wirksame Medikation oder Impfung fehlten, verursachte das neue Virus eine tiefe Verunsicherung in der Bevölkerung. Diese spiegelte sich in einer signifikanten Zunahme der Prävalenz von depressiven Symptomen (Zunahme um 6,7 % auf 14,3 %), Symptomen generalisierter Angst (Zunahme um 10,7 % auf 19,7 %) und allgemeinem Distress (Zunahme um 13,4 % auf 65,2 %) wider [6]. Um die Infektionen einzudämmen, wurden phasenweise vonseiten der Regierungen Kontaktbeschränkungen erlassen und dazu aufgerufen, zwischenmenschliche Kontakte jeglicher Art auf ein absolutes Minimum zu reduzieren. Dies schloss den medizinischen, aber auch den sozialen und Bildungssektor mit ein, sodass die Besuche von Arztpraxen und die Inanspruchnahme ergänzender Therapien, wie Physio- und Ergotherapien, aber auch der Besuch von Kinderbetreuungseinrichtungen und Wohn- und Werkstätten für Behinderte sowie Bildungseinrichtungen gravierenden Veränderungen unterworfen waren.

Menschen mit Seltenen Erkrankungen waren vor dem Hintergrund ohnehin komplexer Versorgungsstrukturen in besonderem Ausmaß von den Veränderungen im medizinischen Sektor, aber auch von den alltäglichen Herausforderungen betroffen. In ihrer Übersichtsarbeit stellen Chowdhury et al. [7] die Belastungen der Betroffenen dar. Über alle Studien hinweg zeigt sich, dass ca. 55 % der Betroffenen Schwierigkeiten in der Inanspruchnahme medizinischer Versorgung erlebten; ca. 70 % berichten vom Ausfall medizinischer Termine. Hintergrund seien vor allem Absagen durch die Praxen oder Klinik gewesen, aber auch Ängste der Betroffenen, sich mit dem Virus zu infizieren. Einer Befragung von EURORDIS, einem europaweiten Zusammenschluss von Patient:innenorganisationen, zufolge seien bei Patient:innen mit systemischer Sklerose selbst Termine wie Chemo- und Hormontherapien ausgefallen [8]. Die Pandemie habe sich aber nicht nur auf die laufende Behandlung von Menschen mit einer Seltenen Erkrankung ausgewirkt, sondern auch auf die Diagnosestellung. Im Zeitraum von Januar bis April 2020 seien in Italien signifikant weniger Neudiagnosen (*n* = 774) als 2019 (*n* = 1272) und 2018 (*n* = 1294) gestellt worden [9]. Die Konsequenzen der verlängerten diagnostischen Odyssee und der veränderten medizinischen Versorgung schlugen sich auch in der wahrgenommenen psychischen Belastung wieder. Circa 55 % aller Befragten nahmen der Übersichtsarbeit von Chowdhury et al. [7] zufolge einen Einfluss der Pandemie auf ihre psychische Gesundheit wahr.

Insbesondere vor dem Hintergrund der massiven Heterogenität der Seltenen Erkrankungen nehmen die Patient:innenorganisationen eine zentrale Rolle ein. Anders als übergeordnete Stellen haben sie die besonderen Bedürfnisse und Anliegen jener Menschen im Blick, die sie vertreten. Sie sind u. a. Informationsquelle und bieten die Möglichkeit, mit anderen Betroffenen in Kontakt zu treten. Neben staatlichen Institutionen und Ärzt:innen sind sie für Betroffene eine wichtige Säule in der Versorgung [10].

Wenngleich die Auswirkungen der Pandemie auf Menschen mit einer Seltenen Erkrankung massiv sind, fehlen für Deutschland Daten zur Situation der Betroffenen. Vor diesem Hintergrund wurde das Forschungsprojekt „Retrospektive Analyse der Versorgungssituation und des Lebensalltags von Menschen mit Seltenen Erkrankungen während einer Pandemie und Ableitung von Handlungsempfehlungen“ (RESILIENT-SE-PAN) durchgeführt, welches von der „Eva Luise und Horst Köhler Stiftung“ gefördert wurde. Im Rahmen des Projekts wurde die Situation von Menschen mit Seltenen Erkrankungen während der Pandemie mithilfe von qualitativen und quantitativen Methoden aus der Betroffenenperspektive näher untersucht. Auf Basis der Ergebnisse wurden Empfehlungen zur Verbesserung der Versorgung von Menschen mit einer Seltenen Erkrankung erarbeitet.

## Methode

**Figure 1:**
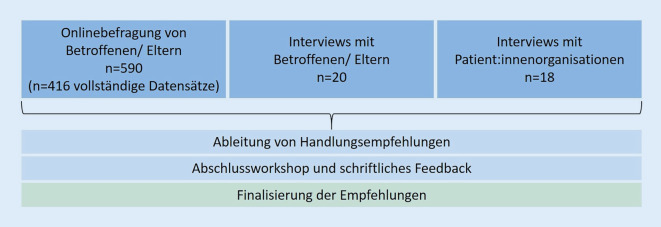

Die Daten wurden im Rahmen einer querschnittlichen Mixed-Methods-Studie erhoben. Dabei wurden quantitative und qualitative Methoden eingesetzt und die Perspektiven der Betroffenen selbst (bzw. der Eltern bei minderjährigen Betroffenen) als auch auf die Perspektive der Patient:innenorganisationen fokussiert. Auf Basis der Ergebnisse wurden zentrale Aspekte identifiziert und in Form von Empfehlungen als Orientierung für mögliche folgende Pandemien formuliert. Ergänzend wurde im Rückmeldeprozess die Perspektive von Versorgenden im Feedbackprozess zu den Empfehlungen miteinbezogen (Abb. 1).

### Datengrundlage

Im ersten Studienteil erfolgte die Erhebung der Betroffenenperspektive auf Grundlage eines Mixed-Methods-Ansatzes. Patient:innen bzw. Eltern bei minderjährigen Patient:innen wurden eingeladen, an einer Onlinebefragung teilzunehmen. Wo möglich, wurden standardisierte Instrumente zur Erfassung relevanter Outcomeparameter (z. B. psychische Befindlichkeit, COVID-spezifische Ängste, Lebensqualität) eingesetzt. Da in Hinblick auf die Auswirkungen der pandemischen Situation keine standardisierten Instrumente vorlagen, wurden diese auf Grundlage der verfügbaren Literatur und der Vorerfahrung sowohl der Forschungsgruppe als auch in enger Abstimmung mit Vertreter:innen von ACHSE e. V. entwickelt. Dabei wurden die Versorgungssituation während der Pandemie, die Auswirkungen auf den Alltag und die Teilhabe sowie Unterstützungsbedarfe erfasst. Die Rekrutierung der Patient:innen bzw. der Eltern erfolgte durch ACHSE e. V. und ihre Mitgliederorganisationen. Darüber hinaus wurden Flyer mit einem QR-Code, der direkt zur Onlinebefragung führte, in Spezialambulanzen von Zentren für Seltene Erkrankungen ausgelegt, um auch Patient:innen zu erreichen, die nicht Mitglied einer Patient:innenorganisation sind. Nach dem Ausfüllen der Onlinebefragung hatten die Teilnehmenden die Möglichkeit, ihr Interesse an einem vertiefenden, semistrukturierten telefonischen Interview anzugeben, für welches ein Wertgutschein in Höhe von 25 € als Aufwandsentschädigung ausgegeben wurde. Die qualitativen Interviews ermöglichten es, ein tiefergehendes Verständnis für die Themenbereiche der Onlinebefragung aufzubauen. Ergänzend wurden Teilnehmende für die vertiefenden Interviews aus bestehenden Interessensbekundungen für eine Studienteilnahme gewonnen, die der Forschungsgruppe durch ein abgeschlossenes Projekt vorlagen [11]. Zur Abbildung eines möglichst umfassenden Erfahrungsspektrums wurde ein Teil der Teilnehmenden auf Grundlage spezifischer Charakteristika wie Geschlecht ausgewählt (Purposive Sampling). Zur Beantwortung deskriptiver Fragestellungen wurde für die Onlinebefragung eine Stichprobengröße von 100–120 Personen angestrebt. Um eine möglichst hohe theoretische Sättigung zu erreichen, wurde für die telefonischen Interviews eine Fallzahl von 15–20 Personen angestrebt [12].

Neben der Betroffenenbefragung erfolgte die Erhebung aus Perspektive der Patient:innenorganisationen. Ebenfalls durch ACHSE e. V. wurden Vertreter:innen von Mitgliedorganisationen zu telefonischen semistrukturierten Telefoninterviews eingeladen. Im Fokus standen die folgenden Themen: Veränderungen der Arbeit in der Patient:innenorganisation durch die Pandemie, Auswirkungen auf die Belastungen und Bedarfe der Betroffenen sowie Wünsche und Empfehlungen für die Versorgung. Es wurde eine Stichprobengröße von 15–20 Interviews angestrebt [12]. Auch in dieser Phase wurde ein Teil der Teilnehmenden auf Grundlage spezifischer Charakteristika ausgewählt (u. a. Geschlecht).

Auf Basis der Ergebnisse beider Projektphasen wurden durch das Projektteam zentrale Aspekte abgeleitet und in Empfehlungen formuliert. Diese wurden im Rahmen eines digitalen Abschlussworkshops diskutiert und anschließend überarbeitet. Am Workshop nahmen neben dem Projektteam 8 Personen teil: 2 Vertreter:innen der ACHSE e. V. und 4 Vertreter:innen von Patient:innenorganisationen. 2 Vertreter:innen der „Eva Luise und Horst Köhler Stiftung“ nahmen als nicht stimmberechtigte Gäste ebenfalls teil. Um auch die Perspektive von Behandelnden mit einzubeziehen, wurden die Empfehlungen durch 2 Ärzt:innen und 2 Psycholog:innen, die im Kontext Seltener Erkrankungen tätig sind, hinsichtlich der Relevanz, Priorisierung und Formulierungen eingeschätzt und ggf. ergänzt oder angepasst. Die Empfehlungen wurden auf Basis der Rückmeldungen erneut durch das Projektteam überarbeitet.

### Auswertung

Zur Beschreibung der Situation der Betroffenen wurden die quantitativen Daten mithilfe deskriptiver Analysen ausgewertet. Für metrische Daten kamen Mittelwert und Standardabweichung zum Einsatz, für kategoriale Daten wurde Häufigkeiten berechnet. Für Untersuchungen von Zusammenhängen zwischen einzelnen Variablen bzw. Konstrukten wurden Korrelationsanalysen eingesetzt.

Das Interviewmaterial wurde transkribiert und durch die Projektmitarbeiter:innen (DZ, MB, LI) ausgewertet. Die Auswertung der qualitativen Analysen erfolgte zum einen mit der qualitativen Inhaltsanalyse nach Mayring [13]. Zum anderen wurde für die Interviews mit Vertreter:innen von Patient:innenorganisationen die Methode des Framework-Ansatzes gewählt [14]. Das biopsychosoziale Modell der Internationalen Klassifikation für Funktionsfähigkeit, Behinderung und Gesundheit (ICF) wurde hierbei als Basis gewählt und die identifizierten Kategorien in die Domänen des Modells integriert [15].

## Ergebnisse

### Stichprobe

#### Betroffene.

Die Onlinebefragung wurde insgesamt 804-mal aufgerufen. Von 590 Menschen wurden mindestens ein Subteil des Fragebogens (z. B. Instrument zur Erfassung Depressivität) sowie Basisangaben zur eigenen Person bzw. Erkrankung ausgefüllt, sodass die Daten in einen Teil der Analysen einbezogen wurden (Minimum Response). 416 Menschen haben den Fragebogen vollständig ausgefüllt.

Alle Teilnehmenden gaben an, an einer Seltenen Erkrankung zu leiden bzw. eine angehörige Person mit einer Seltenen Erkrankung zu haben. Dabei gaben 362 Personen eine Auskunft über ihre Diagnose, während 104 Personen im Rahmen einer anderen Angabe im Fragebogen von der Diagnose berichteten (z. B. Nennung des Selbsthilfevereins). 124 Personen machten keine Angaben zur Diagnose. Die häufigsten Diagnosen bei selbst Betroffenen waren Mukoviszidose (*n* = 60), Sarkoidose (*n* = 49) und angeborener Immundefekt (*n* = 55). Die häufigsten Diagnosen, die von Angehörigen berichtet wurden, waren Mukoviszidose (*n* = 43) und Ösophagusatresie (*n* = 36).

76 % der Teilnehmenden waren Frauen, 1 % hat sich als nicht-binär eingeordnet; 36 % der Befragten waren zwischen 35 und 49 Jahren alt. 142 der 590 Personen haben die Umfrage aus der Perspektive eines Angehörigen (i. d. R. Elternteil) ausgefüllt. 20 Personen nahmen an den vertiefenden telefonischen Interviews teil. Davon waren 12 Personen weiblich; 14 waren selbst von einer Seltenen Erkrankung betroffen.

#### Patient:innenorganisationen.

Insgesamt führte das Studienteam 18 telefonische Interviews mit Vertreter:innen aus 15 unterschiedlichen Patient:innenorganisationen. Die Teilnehmenden waren im Schnitt 55 Jahre alt (Range: 28–72), 12 waren weiblich, 13 Personen hatten den Vorsitz oder zweiten Vorsitz innerhalb ihrer Organisation inne, 2 gehörten dem erweiterten Vorstand an. Eine ausführliche Darstellung der Ergebnisse der Befragung der Patient:innenorganisationen wurde bereits publiziert [16].

### Kernaspekte aus den qualitativen und quantitativen Studienteilen

#### Auswirkungen auf die medizinische Versorgung.

Ein Teil der Betroffenen und Angehörigen berichtete, dass es während der COVID-19-Pandemie zu Ausfällen in der Versorgung oder zur Verschiebung von Terminen kam. Betroffen waren z. B. Behandlungen, Rehabilitationstherapien (Ergo‑, Sprachtherapie o. Ä.), Allgemein- oder Fachärzt:innenbesuche oder diagnostische Untersuchungen. Sowohl die Einrichtungen als auch die Betroffenen selbst initiierten dabei die Absage bzw. Verschiebung. Ein Teil der Studienteilnehmenden schätzte ein, dass die Ausfälle und Verschiebungen negative Auswirkungen auf ihre Gesundheit und das Wohlbefinden hatten. Hinsichtlich der Versorgung in Kliniken oder ähnlichen Einrichtungen berichteten die Teilnehmenden, dass diese teilweise geschlossen gewesen wären, sie diese aufgrund von Ängsten vor einer Infektion nicht aufgesucht hätten oder sie zeitweise angewiesen wurden, die Einrichtung möglichst nicht aufzusuchen. Es wurde von neuen digitalen Angeboten berichtet (z. B. für medizinische Termine, psychologische Betreuung). Teilweise war der Zugang zu Medikamenten, Schutzkleidung, Pflegedienstleistungen und Eingliederungshilfemaßnahmen schwierig.

#### Auswirkungen auf den Lebensalltag.

Hinsichtlich der Anpassungen im Lebensalltag wurden bzgl. des Berufs teilweise Veränderungen wie Homeoffice, flexiblere Arbeitszeiten oder mehr Rückzug von Kolleg:innen genannt. Darüber hinaus wurde von Veränderungen in den Wohnstätten, Schulen und Kitas berichtet. Die pflegerische Versorgung wurde vor allem im Rahmen der allgemeinen Corona-Verordnungen reduziert, zum Teil auch darüber hinaus. Auch Personalmangel der Pflegedienste wurde hier als Grund angegeben. Ein Großteil berichtete von Verringerungen der sozialen Kontakte über die behördlichen Anordnungen hinaus, um das Risiko einer Infektion zu vermeiden. Fehlende Freizeitangebote oder sozialer Austausch wurden berichtet. Als herausfordernd im Alltag wurde erlebt, dass die besondere Situation von Menschen mit einer Seltenen Erkrankung wenig Beachtung fand und es zu Stigmatisierung der Betroffenen kam. Ein Teil der Betroffenen berichtete zudem von finanziellen Auswirkungen durch die Pandemie.

#### Psychische Befindlichkeit.

Als psychisch belastend wurden von den Teilnehmenden u. a. die Isolations- und Einsamkeitsgefühle, ein wahrgenommener Kontrollverlust und Angst vor Ansteckung oder den Auswirkungen einer Infektion erlebt. Eltern erkrankter Kinder berichteten auch von Sorgen über die Auswirkungen der Pandemie bzw. der Schutzmaßnahmen auf die Entwicklung des Kindes. Eltern und Angehörige waren darüber hinaus durch die Pflege- und Versorgungsaufgaben belastet. Die Befragten berichteten depressive und Angstsymptome, die bei einem Teil auf eine moderate bis ausgeprägte Symptomatik hinweisen.

#### COVID-19-spezifische Aspekte.

Die Bedrohlichkeit des Virus wurde von vielen Betroffenen und Angehörigen als groß eingeschätzt. Ein Teil der Befragten begab sich über behördliche Anordnungen hinaus in häusliche Isolation. Viele gaben an, Zugang zu Informationen zu COVID-19 im Allgemeinen zu haben. Spezifische Informationen zu COVID-19 bzw. der entsprechenden Impfung und der Seltenen Erkrankung waren ebenfalls für viele Betroffene und Angehörige zugänglich, jedoch berichtet in der Onlinebefragung etwa jede:r dritte Betroffene, keinen Zugang zu diesen Informationen zu haben. Neben Ärzt:innen, Medien und Internetrecherchen wurden dabei die Patient:innenorganisationen als wichtige Informationsquelle benannt.

#### Unterstützungsbedarfe.

Am häufigsten wurden Unterstützungsbedarfe im psychologischen Bereich, bei alltäglichen Aufgaben (z. B. Haushalt) und Rehabilitationsmaßnahmen berichtet. Bei Eltern bzw. Angehörigen war zudem die Unterstützung durch Pflegeeinrichtungen relevant. Darüber hinaus war die Unterstützung durch Familie, Freund:innen und Nachbar:innen zentral. Von einem Teil der Befragten mit Bedarfen wurde angegeben, dass bereits ausreichende Unterstützung vorliege.

#### Positive Aspekte.

Neben den Herausforderungen wurde über hilfreiche Unterstützungsangebote und zuverlässige Ansprechpartner:innen und Expert:innen in der Versorgung berichtet. Digitale Angebote und Informationsveranstaltungen wurden von vielen Teilnehmenden positiv bewertet. Die Befragten gaben an, dass die gemeinsame Zeit mit der Familie positiv erlebt wurde. Die Patient:innenorganisationen wurden als wichtige Säule in der Versorgung genannt.

### Abgeleitete Empfehlungen

Die im Projekt entwickelten und abschließend abgeleiteten und konsentierten Empfehlungen zur Verbesserung der Versorgung und des Lebensalltags von Menschen mit Seltenen Erkrankungen während einer Pandemie untergliedern sich in 7 verschiedene Bereiche: 1) „medizinische Diagnostik und Versorgung der Seltenen Erkrankung“, 2) „ergänzende Therapien, Hilfs- und Heilmittel“, 3) „Zugang zu Informationen und Impfungen bzgl. COVID-19“, 4) „psychosoziale Unterstützung“, 5) „Alltagshilfen/Teilhabe“, 6) „Patient:innenorganisationen“ und 7) „Sonstige/übergeordnete Aspekte“ (Tab. 1). Für die Bereiche wurden jeweils 1–4 Empfehlungen erstellt. Die Empfehlungen, die sich auf einen direkten persönlichen Kontakt beziehen, sind vor dem Hintergrund einer sorgfältigen Abwägung hinsichtlich des individuellen Infektionsschutzbedarfs zu betrachten und zu gestalten.

**Table 1:** 

**Medizinische Diagnostik und Versorgung der Seltenen Erkrankung**
*Empfehlung 1:*
Die Kontinuität in der Diagnostik und medizinischen Versorgung sollte gewährleistet werden. Die Etablierung und Umsetzung von strukturierten Versorgungspfaden kann die Sicherstellung der Kontinuität unterstützen. Persönliche Kontakte sollten nach dem Infektionsschutzbedarf Betroffener ausgerichtet sein (z. B. Termine am Rand von Sprechstunden, kontaktarme Aufenthaltsmöglichkeiten)
*Empfehlung 2:*
Alternative Versorgungsmodelle, wie z. B. telemedizinische Angebote oder Hausbesuche von Versorgenden, sollten eingesetzt werden, um allen Betroffenen den notwendigen Zugang zu Versorgung zu ermöglichen. Sofern Barrieren in der Nutzung digitaler Angebote aufseiten der Betroffenen vorliegen, sollten persönliche Kontakte umgesetzt werden
*Empfehlung 3:*
Die Versorgung mit und der Zugang zu notwendigen Medikamenten, notwendiger medizinischer Spezialnahrung, Ausrüstung und Schutzkleidung sollte gewährleistet werden
*Empfehlung 4:*
Besuchsregelungen sollten so gestaltet sein, dass der regelmäßige, persönliche, infektionssichere Kontakt zwischen Betroffenen und engen Angehörigen während stationärer Aufenthalte und der Unterbringung in medizinischen und Pflegeeinrichtungen gewährleistet ist. Bei erkrankten Kindern sollten alle Elternteile bzw. Sorgeberechtigten Besuchsrechte während stationärer Aufenthalte und Unterbringung in medizinischen und Pflegeeinrichtungen erhalten
**Ergänzende Therapien, Hilfs- und Heilmittel**
*Empfehlung 1:*
Informationen zu Ansprüchen auf Unterstützungsleistungen (z. B. durch Kranken- und Pflegeversicherungen), inkl. Hinweise zur Beantragung und Inanspruchnahme, sollten barrierefrei zugänglich und zur Verfügung gestellt werden sowie in laienverständlicher Sprache formuliert sein und in verschiedenen Sprachen vorliegen
*Empfehlung 2:*
Die Kontinuität ambulant-therapeutischer Angebote (inkl. Ergotherapie, Logopädie, Frühförderung, Physiotherapie etc.) sowie die Ausstattung mit Hilfsmitteln (z. B. Hörgeräte, Brillen, Prothesen) sollten gewährleistet werden und im persönlichen Kontakt (z. B. bei den Betroffenen zuhause) stattfinden, sofern der Nutzen des persönlichen Kontakts in Abwägung des Risikos einer Infektion und Weiterverbreitung des Krankheitserregers gegeben ist. Die Möglichkeit der Umsetzung dieser Angebote in telemedizinischer Form kann im Einzelfall, z. B. auf Wunsch der betroffenen Person, geprüft werden, sollte aber in jedem Fall barrierefrei sein
*Empfehlung 3:*
Bei Veränderungen der Strukturen im Gesundheitssystem und damit verbundenen Herausforderungen in der Gesundheitsorganisation besteht eine besondere Dringlichkeit nach einer unterstützenden Koordinierungsstelle/-person
**Zugang zu Informationen und Impfungen bzgl. COVID-19**
*Empfehlung 1:*
Deutsche und europäische Referenznetzwerke sollten ihre Strukturen nutzen, um a) zeitnah qualitätsgesicherte Informationen zu den Auswirkungen der COVID-19-Erkrankung auf die von ihnen vertretenen Erkrankungsgruppen bereitzustellen und b) nationale und internationale Daten/Register zur Generierung von Evidenz zu Auswirkungen einer COVID-19-Erkrankung und/oder Nebenwirkungen der Impfung zu erfassen
Die Informationen sollten proaktiv an die Betroffenen weitergeleitet werden, barrierefrei zugänglich sein und in laienverständlicher Sprache sowie ggf. in der Muttersprache der Betroffenen formuliert sein. Als Multiplikatoren können z. B. Fachärzt:innennetzwerke oder Patient:innenorganisationen genutzt werden, um die Informationen an ihre Patient:innen/Mitglieder weiterzuleiten. Eine Bereitstellung der Informationen im SE-Atlas, als zentrale Struktur für die Bereitstellung und Verbreitung von Informationen, ist ebenfalls denkbar. Hierzu ist eine stärkere Förderung des SE-Atlas notwendig
*Empfehlung 2:*
Sofern durch eine Infektion ein erhöhtes gesundheitliches Risiko zu erwarten ist, sollte die Möglichkeit zur prioritären Impfung der Menschen mit Seltenen Erkrankungen sowie ihrer pflegenden Angehörigen gewährleistet und der Zugang zur Impfung barrierefrei gestaltet sein. Informationen dazu sollten durch eine zentrale Informationsstelle vorgehalten werden, die ggf. auf regionale Ansprechpartner:innen verweisen kann
**Psychosoziale Unterstützung**
*Empfehlung 1:*
Psychosoziale, sozialrechtliche und pädagogische Unterstützungsangebote für Betroffene und Angehörige sollten bedarfsorientiert und barrierefrei, ggf. auch in telemedizinischer Form, zur Verfügung stehen. Eine Übersicht über mögliche Anlaufstellen und Ansprechpartner:innen (z. B. Sozialberatungen, psychosoziale Beratungsstellen, ergänzende unabhängige Teilhabeberatung – EUTB®) sollte barrierefrei zur Verfügung stehen
*Empfehlung 2***:**
Psychosozialer Konsil- oder Liaisondienst in den Kliniken sollte bedarfsorientiert auch für Betroffene mit Seltenen Erkrankungen und ihre Angehörigen bereitgestellt werden
*Empfehlung 3:*
Ärzt:innen sollten Betroffene und Angehörige bei Bedarf an psychosoziale Unterstützungsangebote und an Patient:innenorganisationen weiterleiten
**Alltagshilfen/Teilhabe**
*Empfehlung 1***:**
Pflegedienstleistungen sollten kontinuierlich gewährleistet werden
*Empfehlung 2:*
Die Kontinuität der Betreuung, soziale Teilhabe und Bildung (Schule, Kindergarten, Krippe, Hort etc.) sollte für Kinder mit Seltenen Erkrankungen gewährleistet werden und nach dem Infektionsschutzbedarf Betroffener ausgerichtet sein (z. B. Hygienekonzepte). Zusätzliche, flexible Entlastungsangebote für Eltern sollten geschaffen werden
*Empfehlung 3:*
Die Kontinuität der Leistungen zur medizinischen Rehabilitation, zur Teilhabe an Bildung und Arbeit und zur sozialen Teilhabe (inkl. Tagesförderstätten, arbeitstherapeutischer Maßnahmen, Berufsbildungswerke, Assistenzleistungen, Transportmöglichkeiten etc.) sollte gewährleistet werden und an dem Infektionsschutzbedarf Betroffener ausgerichtet sein
*Empfehlung 4:*
Die Möglichkeit zur gesellschaftlichen Teilhabe unter Sicherstellung des Infektionsschutzes (z. B. öffentliche Einrichtungen, ÖPNV, Veranstaltungen, Gruppenangebote) für Betroffene und Angehörige sollte gewährleistet werden (z. B. separate Schutzbereiche)
**Patient:innenorganisationen**
*Empfehlung 1:*
Die Arbeit der Patient:innenorganisationen sollte nachhaltig unterstützt werden, da sie als niederschwellige Ansprechpartner:innen der Betroffenen und ihrer Angehörigen vielfältige Aufgaben übernehmen
**Sonstige/übergeordnete Aspekte**
*Empfehlung 1:*
Antragsverfahren und die Organisation der Versorgung und Teilhabe sollten entbürokratisiert werden. Informationen dazu sollten barrierefrei und in laienverständlicher Sprache sowie in unterschiedlichen Sprachen zur Verfügung stehen
*Empfehlung 2:*
Die Öffentlichkeit, Behörden und Institutionen sollten für die Situation der Betroffenen während der Pandemie und die noch immer bestehenden Belastungen sensibilisiert werden
*Empfehlung 3:*
Menschen mit Seltenen Erkrankungen als vulnerable Gruppe sollten bei (gesundheits-)politischen Entscheidungen, die sich auf ihre Versorgung und ihren Schutz vor Infektionen auswirken, berücksichtigt werden

## Diskussion

Die COVID-19-Pandemie sowie die damit verbundenen Maßnahmen zur Eindämmung hatten Auswirkungen auf die gesamte Bevölkerung in Deutschland. Als besonders vulnerable Gruppe waren Menschen mit Seltenen Erkrankungen auf unterschiedliche Weise betroffen. Je nach Grunderkrankung galten bzw. gelten sie als Risikogruppe im Falle einer Infektion mit COVID-19. Im Rahmen des Projekts RESILIENT-SE-PAN wurde daher die Situation von Menschen mit Seltenen Erkrankungen bzw. deren Angehörigen (i. d. R. Eltern) untersucht und es wurden Empfehlungen abgeleitet, um im Falle zukünftiger Pandemien oder Krisen des Versorgungs- und Gesundheitssystems für die Situation dieser Menschen zu sensibilisieren und zu beachtende Aspekte vorzugeben. Bei der Untersuchung zeigten sich Auswirkungen der Pandemie auf die Versorgung, den Lebensalltag und die Psyche der Betroffenen. Die durchschnittliche psychische Belastung der Teilnehmenden war höher als in der Allgemeinbevölkerung [17].

Die Sicherstellung der medizinischen Diagnostik und Versorgung ist ein zentraler Punkt innerhalb der Empfehlungen. Telemedizinische Angebote bieten hier v. a. auch für verunsicherte Betroffene oder jene mit hohem Infektionsschutzbedarf die Möglichkeit, weiter in Kontakt mit ihren Versorgenden zu bleiben [18]. Es ist dabei aber zu beachten, dass ältere Menschen oder Menschen mit starken Beeinträchtigungen über diese Angebote ggf. nicht erreicht werden können. Auch die Aufrechterhaltung und der niederschwellige Zugang zu ergänzenden Therapien, Hilfs- und Heilmitteln, Informationen und psychosozialer Unterstützung sind während einer Pandemie unerlässlich. In einer 2020 durchgeführten Befragung gaben bis zu 46 % der Teilnehmenden an, dass der Wegfall unterstützender Angebote eine Verschlechterung ihres Gesundheitszustandes nach sich zog [19]. Auch in der vorliegenden Befragung wurden Auswirkungen auf die Gesundheit und das Wohlbefinden berichtet. Infolge der Lockdowns erlebten einer anderen Umfrage aus dem Vereinigten Königreich und Irland zufolge 56 % der Befragten mit Glasknochenerkrankung (Osteogenesis imperfecta) Mobilitätseinschränkungen und 72 % vermehrt Schmerzen [20]. Mangelnde Kenntnis über Unterstützungsleistungen erschwerte den Zugang ebenfalls. Verunsicherungen und COVID-bezogene Ängste können durch fehlende Informationen zu COVID, den Auswirkungen einer Infektion oder zur Indikation für eine Impfung verstärkt werden. Hinsichtlich des Alltags und der gesellschaftlichen Teilhabe zeigten die Ergebnisse auf der einen Seite die Notwendigkeit der Kontinuität von Angeboten und Dienstleistungen. Gleichzeitig wurde deutlich, dass die Möglichkeit der Teilhabe auch bei Vorliegen eines erhöhten Bedarfs an Infektionsschutz für die Betroffenen gewährleistet sein sollte (z. B. durch Maske tragen, Abstandsregelungen). Wie sich sowohl in unserer als auch internationalen Studien gezeigt hat [10], übernehmen die Patient:innenorganisationen eine wichtige Rolle bei der Unterstützung der Betroffenen. Die übergeordneten Empfehlungen beziehen sich v. a. auf die Sensibilisierung und Beachtung der Situation von Menschen mit Seltenen Erkrankungen.

Während in vielen bisherigen Studien übergeordnete Parameter (z. B. Lebensqualität, Angst) zur Erfassung der Situation von Menschen mit Seltenen Erkrankungen bzw. ihren Angehörigen eingesetzt wurden, basieren die vorliegende Studie und die abgeleiteten Empfehlungen auf Daten, die explizit die Auswirkungen der Pandemie aus Betroffenenperspektive erfasst haben. Da die Erhebung im Sommer und Herbst 2022 stattfand, konnten die teilnehmenden Personen eine umfassende Rückschau auf mehr als 2 Jahre Pandemie vornehmen und über die im Verlauf der Pandemie erlebten Veränderungen berichten. Neben den Erfahrungen der Betroffenen wurde die Perspektive der Patient:innenorganisationen erfasst, die in gebündelter Form die Erfahrungen ihrer Mitglieder schilderten. Dies erlaubt es, Empfehlungen zu formulieren, die den Verlauf der Pandemie (Lockdown, Impfungen, Lockerungen) mit aufgreifen. Die abgeleiteten Empfehlungen können durch internationale Befunde gestützt und unterstrichen werden: Im Kontext der Situation von Menschen mit Seltenen Erkrankungen wurden COVID-spezifische Ängste [21–23] und Unterbrechungen in der Versorgung und Diagnostik berichtet [9, 24] sowie auf den Bedarf nach psychosozialen Unterstützungsangeboten [25–27] hingewiesen.

Folgende Limitationen müssen bei der Betrachtung der abgeleiteten Empfehlungen beachtet werden: Da es sich um eine freiwillige Teilnahme handelte, liegt ein Selektionseffekt vor. Die Krankheitsbilder der Betroffenen, die an der Onlinebefragung teilgenommen haben, bilden nicht das komplette Spektrum der Seltenen Erkrankungen ab, sondern konzentrieren sich auf ausgewählte Diagnosegruppen, die während der COVID-19-Pandemie möglicherweise besondere Einschränkungen erlebt haben. Da die Information über die Studienteilnahme fast vollständig über die Selbsthilfe bzw. Patient:innenorganisationen umgesetzt wurde, ist die Perspektive von Menschen, die nicht an einer solchen Organisation angebunden sind, unterrepräsentiert. Darüber hinaus fehlte in der Datenerhebung die Perspektive von Vertreter:innen der klinischen Versorgung sowie in der Diskussion der Empfehlungen eine Vertretung niedergelassener Kinder‑, Fach- oder Allgemeinärzt:innen. Es ist somit insbesondere die Perspektive der Betroffenen widergespiegelt.

Es ist zu beachten, dass die Empfehlungen auf Basis der Ergebnisse ausschließlich eine Orientierungshilfe für die Gestaltung des Gesundheitssystems in Krisenzeiten darstellen können. Eine mögliche Umsetzung in vorhandenen Strukturen oder mögliche Veränderungen der Strukturen werden dabei nicht weiter vertieft und wären zu entwickeln. Während ein Teil der Empfehlungen spezifisch mit den Herausforderungen und der Situation von Menschen mit Seltenen Erkrankungen verknüpft ist, können einige Empfehlungen auch auf andere schwere, chronische Erkrankungen übertragen werden. Insgesamt ist hinsichtlich der abgeleiteten Empfehlungen zu beachten, dass für die Umsetzung sowohl strukturelle Voraussetzungen einzelner Einrichtungen als auch die Finanzierung der Umsetzung gewährleistet werden muss. Auch die Abwägung zwischen dem Risiko einer Infektion und dem Bedarf an direktem persönlichen Kontakt erfordert ggf. individuelle Lösungen, die im Einzelfall und situationsspezifisch angepasst werden müssen.

## Fazit

Während der Coronapandemie erlebten Menschen mit Seltenen Erkrankungen bzw. ihre Angehörigen starke Belastungen und Verunsicherungen. Neben der Aufrechterhaltung der medizinischen Versorgung und der therapeutischen Angebote sind auch der Zugang zu Informationen, psychosozialer Unterstützung und Alltagshilfen zentral. Das beschriebene Projekt konnte hierzu auf Basis qualitativer und quantitativer Daten Empfehlungen entwickeln, die eine Orientierung liefern können, welche Aspekte in Krisen des Gesundheitssystems (z. B. Pandemien) für die Versorgung von Menschen mit Seltenen Erkrankungen zu beachten sind.
